# High prevalence of *Blastocystis* sp. in farmed sika deer (*Cervus nippon*) in Northern China

**DOI:** 10.3389/fvets.2025.1562814

**Published:** 2025-04-07

**Authors:** Zhen-Qiu Gao, Yang Gao, Hong-Di Zhuang, Guang-Rong Bao, Jing Liu, Jian-Ming Li, Nian-Yu Xue, Hong-Wei Cao, Shuo Liu

**Affiliations:** ^1^School of Pharmacy, Yancheng Teachers University, Yancheng, Jiangsu Province, China; ^2^College of Life Sciences, Changchun Sci-Tech University, Shuangyang, Jilin Province, China; ^3^State Key Laboratory for Animal Disease Control and Prevention, Harbin Veterinary Research Institute, Chinese Academy of Agricultural Sciences (CAAS), Harbin, Heilongjiang Province, China; ^4^College of Veterinary Medicine, Jilin Agricultural University, Changchun, Jilin Province, China; ^5^College of Veterinary Medicine, Qingdao Agricultural University, Qingdao, Shandong Province, China; ^6^College of Veterinary Medicine, Yangzhou University, Yangzhou, Jiangsu Province, China

**Keywords:** *Blastocystis* sp., sika deer, prevalence, zoonotic, China

## Abstract

**Introduction:**

*Blastocystis* sp. is a widespread intestinal protist, that threatens the health of humans and animals. However, epidemiological data on *Blastocystis* sp. in sika deer are still scarce in China and globally. This study aimed to reveal the infection rate, and subtype distribution of *Blastocystis* sp. in farmed sika deer across China.

**Methods:**

A total of 466 fresh fecal samples were collected from farmed sika deer in northern China. A 600 bp fragment of the SSU rRNA was amplified to detect the presence of *Blastocystis* sp. in samples.

**Results:**

The results revealed an overall infection rate of *Blastocystis* sp. at 65.02% (303/466). The highest infection rate was found in Shandong (98%, 49/50), followed by Heilongjiang (95.51%, 85/89), while the lowest infection rate was found in Jilin (36.31%, 61/168). Additionally, within the seasonal group, the infection rate was significantly higher in autumn (69.04%) than in summer (43.06%). In the age group, although the infection rate in young animals (68.38%) was higher than in adults (63.90%), no statistical difference was observed. Ten subtypes were identified from 303 *Blastocystis* sp.-positive samples, including ST1, ST5, ST10, ST14, ST21, ST23, ST24, ST26, ST30 and ST42. Among these, ST10 was the dominant subtype with an infection rate of 43.23% (131/303), and ST1 and ST5 were zoonotic subtypes. Notably, this study is the first to report the presence of ST42 in sika deer.

**Discussion:**

These findings suggest that sika deer may be a potential source of human *Blastocystis* sp. infection. In addition, this study reveals the high infection rate of *Blastocystis* sp. in farmed sika deer and reports for the first time the presence of ST42 subtype, providing valuable data for the epidemiological study of *Blastocystis* sp. in sika deer.

## Introduction

*Blastocystis* sp. is a common single-celled intestinal protist that is primarily transmitted through the fecal-oral route and can colonize the gastrointestinal tract of humans, domestic animals and wild animals (1). The parasite is widely distributed around the world, especially in low income countries with high infection rates. For example, the prevalence of *Blastocystis* sp. infection among residents in Laos was 41.7%; the infection rate among schoolchildren in Ecuador was 39.2% (2, 3). At present, the pathogenicity of *Blastocystis* sp. has not been conclusively concluded (1). On the one hand, *Blastocystis* sp. exists in people with normal immune function and HIV or diabetes patients and manifests as asymptomatic (1, 4, 5). On the other hand, studies have shown that *Blastocystis* sp. can produce cysteine protease, which can cause host inflammatory response and gastrointestinal tissue damage, and show vomiting, diarrhea, abdominal pain and other symptoms (6).

*Blastocystis* sp. has a rich morphological and genetic diversity (7). Because it is difficult to distinguish different subtypes by morphology, researchers used PCR technology to analyze the sequence of small subunit ribosomal RNA (*SSU* rRNA), thereby revealing its genetic diversity (8). Currently, about 44 *Blastocystis* sp. subtypes have been identified (9). The subtypes that humans can carry include ST1-ST10, ST12-ST14, ST16, ST35 and ST41 (10). Among them, ST1-ST4 is the most common, accounting for 90% of *Blastocystis* sp. infections in humans (11). Additionally, the ST1-ST4 subtypes have been reported in other animals such as non-human primates and rodents (12, 13). ST9 was originally only found in humans, but it has also been reported in peacocks (*Pavo cristatus*) in China (14, 15). ST10 and ST14 have been found in ruminants such as camels and sheep (16, 17). These findings suggest that there may be more unknown species hosts for the subtypes associated with human infections, and therefore more studies are needed to reveal the host range of different *Blastocystis* sp. subtypes.

*Blastocystis* sp. infection has been reported in ruminants around the world, especially in China (17). For example, the infection rate was 12.60% in cattle in Hebei Province, 7.5% in Tibetan sheep (*Pantholops hodgsonii*) in Qinghai Province, and 10.70% in sheep in Inner Mongolia Autonomous Region, among others (9, 18, 19). Sika deer is widely distributed in the East Asian mainland, the Japanese islands and parts of Europe (20). As an important economic animal, it is often raised for meat and tonic production. In China, sika deer have a long history of domestication. Over the past few decades, the sika deer farming industry has steadily developed, with the population increasing from 100,000 in 1950 to 1.2 million today, accounting for more than 90% of the global farmed population (21). However, research on *Blastocystis* sp. infection in sika deer is still limited in China and even globally. This study aimed to determine the infection rate and distribution of *Blastocystis* sp. in farmed sika deer in northern China to enrich the data on *Blastocystis* sp. in sika deer.

## Materials and methods

### Sample collection

From May to October 2024, a total of 466 fresh fecal samples from sika deer were collected using convenience sampling from farms in Jilin (*n* = 168), Heilongjiang (*n* = 59), Liaoning (*n* = 159), and Shandong (*n* = 50), China. To enhance representativeness, sampling was conducted across the entire farm rather than a specific location. All samples were directly collected by researchers. Each sample was collected with disposable PE gloves and into a sterilized sampling tube. The samples were labeled with sampling location, and time and then transported to the laboratory on dry ice and stored at −80°C until extract DNA.

### DNA extraction and PCR amplification

200 mg of each fecal sample was taken into a 2 mL centrifuge tube with 200 mg glass beads, DNA was extracted with an E.Z.N.A.® Stool DNA Kit (Omega Bio-Tek Inc., Norcross, GA, United States) according to the manufacturer’s instructions. 200 μL elution buffer was taken into each sample to elute DNA and stored at −20°C until PCR amplification.

Forward primer RD5: 5’-ATCTGGTTGATCCTGCCAGT-3′ and reverse primer BhRDr: 5’-GAGCTTTTTAACTGCAACAACG-3′ were used to amplify a 600 bp fragment of *SSU* rRNA for detecting the presence of *Blastocystis* sp. in samples (22). A 30 μL mixture consists of 15 μL of 2 × Specific™ Taq Master Mix (Quick Load, Novoprotein Technology Co., Ltd., Shanghai, China), 11 μL of double-distilled water (ddH_2_O), 1 μL each of forward and reverse primer (10 μ*M*), respectively, and 2 μL of DNA template. PCR amplification conditions were as follows: predenaturation at 94°C for 5 min, denaturation at 94°C for 1 min, annealing at 59°C for 1 min, extension at 72°C for 1 min, repeated for 35 cycles, and a final extension at 72°C for 3 min. The PCR process includes a negative control and a positive control. PCR products were analyzed by 1% agarose gel electrophoresis, and visualized on QuickGel 6200 (Monad Biotech Co., Ltd., Hubei, China).

### Sequence analysis

All PCR products of *Blastocystis* sp.-positive were sent to Welai Biotech Co., Ltd. (Qingdao, China) for bidirectional sequencing. Basic Local Alignment Search^1^ was used to align the assemblage sequences with reference sequences in the GenBank database. CD-HIT with a threshold set to 0.99 was used to cluster the sequences in the present study. PubMLST^2^ was conducted to identify the subtypes of *Blastocystis* sp. in the present study.

### Phylogenetic analysis and nucleotide sequence accession numbers

The ClustalW algorithm in the MEGA11 was used to align 27 representative sequences from this study and 21 reference sequences from the GenBank. The phylogenetic analysis was conducted by neighbor-joining (NJ) methods and the Kimura 2-parameter model. 1,000 bootstrap replications were performed to assess the stability of the results.

The representative sequences in this study were submitted to the GenBank database, with the accession numbers: PQ817670-PQ817696.

### Statistical analysis

A chi-square was conducted in SAS (v. 9.4) to examine the differences in infection of *Blastocystis* sp. among the regions (*x*1) season (*x*2) and age (*x*3). Fisher’s scoring method was used to judge the best model. The infection rates with 95% confidence intervals (95% CI) and odds ratio (OR) were calculated by the Wald method in the SPSS 26.0 version (SPSS Inc., Chicago, IL, United States). All tests were two-sided and a *p*-value less than 0.05 was considered statistically significant.

## Results

### Infection rates of *Blastocystis* sp. in sika deer

The overall infection rate of *Blastocystis* sp. was 65.02% (303/466, 95% CI 60.50–69.35) in farmed sika deer in China. There was a significant difference in the infection rate between regions (*χ*^2^ = 158.09, df = 3, *I*^2^ = 98.1 *p* < 0.0001) ranging from 38.69 to 98.00%. Among these, the highest infection rate was observed in Shandong (98.00%, 49/50, 95% CI 91.61–100.00), followed by Heilongjiang (95.51%, 85/89, 95% CI 90.01–99.02). Jilin showed the lowest infection rate at 38.69% (61/168, 95%CI 29.19–43.75) There were also significant differences in the infection rate in the seasonal groups (*χ*^2^ = 17.01, df = 11, *I*^2^ = 94.1 *p* < 0.0001), and the infection rate in autumn (69.04%, 272/394) was significantly higher than that in summer. Additionally, there were little difference in infection rates between adult (63.90%, 223/349) and young (68.38%, 80/117) animals (Table 1).

**Table 1:** Prevalence of *Blastocystis* sp. infection rate in sika deer by different regions.

Factors	Category	No. tested	No. positive	% (95% CI)	Heterogeneity	OR (95%CI)
					*χ*^2^/df/*I*^2^(%)/P	
Regions	Jilin	168	61	36.31(29.19–43.75)	158.09/3/98.1/<0.0001	Reference
	Heilongjiang	89	85	95.51 (90.01–99.02)		37.28 (13.03–106.62)
	Liaoning	159	108	67.92 (60.44–74.98)		3.72 (2.35–5.87)
	Shandong	50	49	98.00 (91.61–100.00)		85.95 (11.58–638.07)
Season	Summer	72	31	43.06 (31.78–54.69)	17.01/1/94.1/<0.0001	Reference
	Autumn	394	272	69.04 (64.37–73.51)		2.95 (1.77–4.93)
Age	Adult	349	223	63.90 (58.78–68.86)	0.75/1/0/0.3873	Reference
	Young	117	80	68.38 (59.63–76.52)		1.22 (0.78–1.91)
Total	–	466	303	65.02 (60.50–69.35)	–	–

### Distribution of subtypes

This study identified 10 subtypes of *Blastocystis* sp., including ST1 (*n* = 95), ST5 (*n* = 7), ST10 (*n* = 131), ST14 (*n* = 8), ST21 (*n* = 30), ST23 (*n* = 2), ST24 (*n* = 18), ST26 (*n* = 4), ST30 (*n* = 5), and ST42 (*n* = 3). Among them, ST10 accounted for 43.23% (131/303) of the infection and was the dominant subtype in farmed sika deer in China. Notably, ST1 is the known zoonitic subtype with a high infection at 31.35% (95/303) (Table 2).

**Table 2:** Distribution of *Blastocystis* sp. subtypes in sika deer in China.

Factors	Category	Subtypes
Regions	Jilin	ST1 (*n* = 16), ST10 (*n* = 29), ST14 (*n* = 3), ST21 (*n* = 7), ST24 (*n* = 2), ST26 (*n* = 3), ST30 (*n* = 1)
	Heilongjiang	ST1 (*n* = 22), ST5 (*n* = 3), ST10 (*n* = 31), ST14 (*n* = 2), ST21 (*n* = 20), ST24 (*n* = 7)
	Liaoning	ST1 (*n* = 57), ST5 (*n* = 3), ST10 (*n* = 35), ST14 (*n* = 3), ST21 (*n* = 1), ST23 (*n* = 2), ST24 (*n* = 3), ST30 (*n* = 4)
	Shandong	ST5(*n* = 1), ST10 (*n* = 36), ST21 (*n* = 2), ST24 (*n* = 6), ST26 (*n* = 1), ST42 (*n* = 3)
Season	Summer	ST1(*n* = 14), ST10 (*n* = 15), ST14 (*n* = 1)
	Autumn	ST1 (*n* = 85), ST5 (*n* = 7), ST10 (*n* = 116), ST14 (*n* = 7), ST21 (*n* = 30), ST23 (*n* = 2), ST24 (*n* = 18), ST26 (*n* = 4), ST30 (*n* = 5)
Age	Adult	ST1 (n = 29), ST5 (*n* = 5), ST10 (n = 26), ST14 (*n* = 5), ST21 (*n* = 1), ST23 (*n* = 2), ST24 (*n* = 8), ST30 (*n* = 4)
	Young	ST1 (*n* = 66), ST5 (*n* = 2), ST10 (*n* = 105), ST14 (*n* = 3), ST21 (*n* = 29), ST24 (*n* = 10), ST26 (*n* = 26), ST30 (*n* = 1)
Total		ST1 (*n* = 95), ST5 (*n* = 7), ST10 (*n* = 131), ST14 (*n* = 8), ST21 (*n* = 30), ST23 (*n* = 2), ST24 (*n* = 18), ST26 (*n* = 4), ST30 (*n* = 5), ST42 (*n* = 3).

In this study, ST1, ST10, and ST14 were found in Jilin, Heilongjiang, and Liaoning provinces. Except for Jilin, ST5 had a small distribution in the other provinces. Additionally, ST21 and ST24 were distributed in all four provinces, while ST42 was only found in Shandong and ST23 exclusively in Liaoning. ST30 was detected in both Jilin and Liaoning, and ST26 was found in both Jilin and Shandong.

### Risk factors

In the present study, logistic forward stepwise analysis and Fisher’s scoring method were used to evaluate the influence of factors on the infection rate of *Blastocystis* sp. in sika deer. The result showed that region and season had a negative impact on the infection rate of *Blastocystis* sp., as described by the equation: *y* = −5.92*×*1-1.14*×*2 + 3.51. The infection rate is highest in Shandong and lowest in Jilin. Additionally, sika deer are more susceptible to *Blastocystis* sp. infection in autumn compared to in summer.

### Phylogenetic analysis

**Figure 1:**
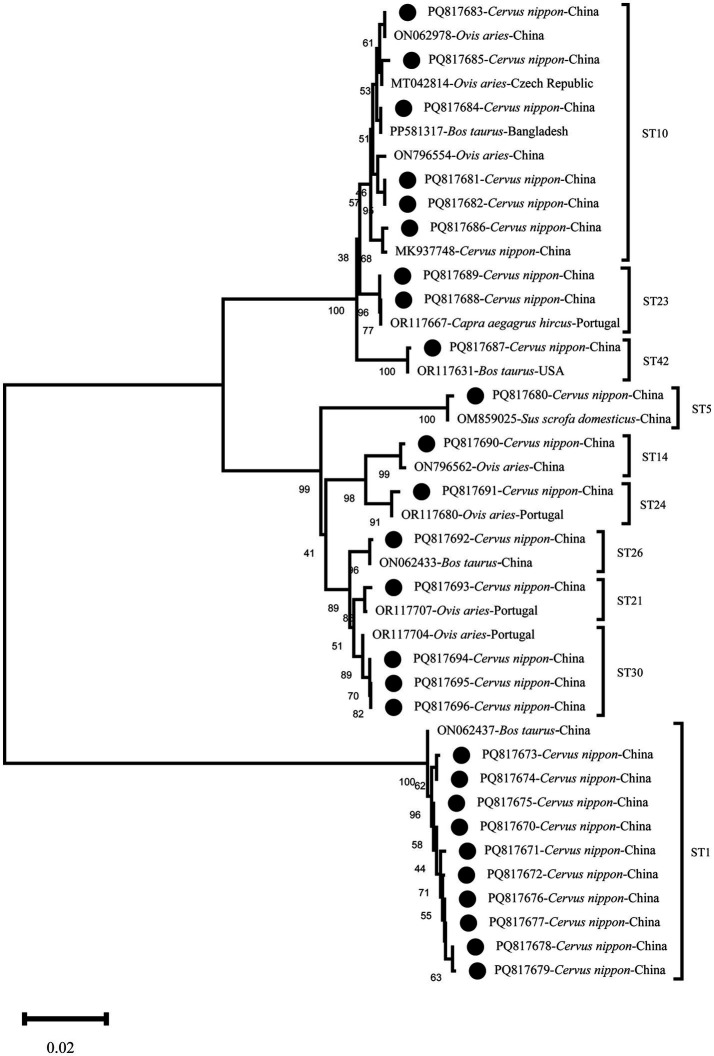
Phylogenetic relationships of *Blastocystis* sp. subtypes based on sequences of the partial small subunit ribosomal RNA (*SSU* rRNA) gene. The neighbor-joining (NJ) method was used to construct the trees from the Kimura-2-parameter model. Branch numbers represent per cent bootstrapping values from 1,000 replicates, with values of more than 50% shown in the tree. *Blastocystis* sp. found in the present study are marked with black dots on the tree.

The results of the phylogenetic tree analysis showed that the representative sequences of this study clustered with the reference sequences in their corresponding subtype branches. Specifically, PQ817670-PQ817679 clustered as sister branches withON062437 (Cattle, China), exhibiting a similarity of 99.6–100%; PQ817680 showed 100% similarity with OM859025 (Pig, China), clustering within the ST5 branch. In the ST10 branch, PQ817681 and PQ817682 showed 99.7% similarity with ON796554 (Sheep, China), PQ817683 showed 100% similarity with ON062978 (Sheep, China), PQ817684 matched PP581317 (Cattle, Bangladesh) with 100% similarity, PQ817685 showed 99.8% similarity with MT042814 (Sheep, Czech Republic), and PQ817686 showed 100% similarity with MK937748 (Muntjac, China). PQ817687 showed 100% similarity with OR117631 (Cattle, United States), clustering within the ST42 branch. In the ST23 branch, PQ817688 showed 100% similarity with OR117667 (Goat, Poland) and formed a sister branch with PQ817689. In the ST14 and ST24 branches, with a bootstrap value of 98%, PQ817690 showed 99.8% similarity with ON796562 (Sheep, China), PQ817691 showed 100% similarity with OR117680 (Sheep, Portugal), PQ817692 showed 100% similarity withON062433 (Cattle, China), PQ817693 showed 100% similarity with OR117707 (Sheep, Portugal), and PQ817694-PQ817696 showed 99.8% similarity with OR117704 (Sheep, Portugal), clustering within the same branch (Figure 1).

## Discussion

The present study found that the overall infection rate of *Blastocystis* sp. in sika deer was 65.02% (303/466), significantly higher than previously reported in other regions of China. For example, the infection rate in the Tibetan Plateau was 0% (0/8), in the Northeast was 14.6% (12/82), and in the northern regions was only 0.8% (6/760) (23–25). Additionally, the infection rates in other cervid species in China were generally lower than those observed in this study, such as in white-lipped deer (*Cervus albirostris*) (50%, 1/2), reindeer (*Rangifer tarandus*) (6.73%, 7/104), Alpine musk deer (*Moschus chrysogaster*) (39.8%, 80/201), forest musk deer (*Moschus berezovskii*) (14.7%, 74/504), and Père David’s deer (*Elaphurus davidianus*) (56.3%, 72/128) (24, 26–29). Studies in neighboring countries also reported lower infection rates, including in Japanese sika deer (45.5%, 60/132) and Korean water deer (*Hydropotes inermis*) (40.8%, 51/125) (30, 31). These differences may be attributed to various factors, including sampling time, geographical environment, farming conditions, and species differences.

In this study, region was one of the risk factors for *Blastocystis* sp. infection. The infection rates ranged from 36.31 to 98%, with the highest infection rate observed in Shandong (98.00%, 49/50), followed by Heilongjiang (95.51%, 85/89), and the lowest infection rate was found in Liaoning at 36.31% (61/168). These differences in infection rates may be related to stocking density, management measures and sample size. Season was another risk factor, sika deer seemed to be more susceptible to *Blastocystis* sp. infection in autumn (69.04%) than in summer (43.06%), this result was similar to a study on *Blastocystis* sp. infecton in dogs in Korea (32). This seasonal difference may be related to temperature and humidity changes that weaken the immune system of the animals. Additionally, the infection rate in young animals (68.38%) was slightly higher than that in adults (63.90%) in this study. This phenomenon is consistent with the current epidemicology data (33). The higher rate of infection in young animals may be mainly due to the fact that their immune systems are not fully developed, resulting in less resistance and greater susceptibility to pathogen attack. Additionally, physiological characteristics of young animals, such as higher metabolic rates and unique hormone levels, may also play an important role in infection rate differences.

In this study, 10 subtypes of *Blastocystis* sp. were identified from 303 positive samples, including ST1, ST5, ST10, ST14, ST21, ST23, ST24, ST26, ST30, and ST42.ST10 was the predominant subtype in sika deer, further supporting its role as the primary subtype in animal infections (34). Additionally, ST1, ST14, ST21, ST23, ST24, ST26, and ST30 have been reported in white-tailed deer (*Odocoilrus virginianus*) in the United States, while ST1 and ST14 have also been found in alpine musk deer and forest musk deer in China, indicating the significant presence of these two subtypes in *Blastocystis* sp. infections in cervids (26, 29, 35). ST5 has been recorded in European roe deer (*Capreolus capreolus*) in the United Kingdom, forest musk deer in China, and red brocket (*Mazama americana*) in Brazil (36, 37). Notably, ST42 appears to be the first report of this genotype in cervids, it has previously been detected in herbivorous animals in Portugal (38).

Epidemiological data show that ST1 is widely distributed across rodents, ruminants, and primates, including humans, demonstrating low host specificity and strong cross-host transmission ability, while exhibiting significant zoonotic characteristics (39). In this study, 10 representative ST1 sequences showed up to 100% similarity with the reference sequence KY675364 (human, Turkey), indicating that sika deer may be potentially at risk for transmitting zoonotic subtypes. Furthermore, ST5 is also an important zoonotic subtype, widely distributed in pigs, cattle, sheep, pet dogs and other animals in close contact with humans (17, 32, 40). A study in Thailand has shown that ST5 is the dominant subtype of transmission between breeders and pigs in pig farms, and close contact may lead to the transmission of ST5 (41). Therefore, special attention should be paid to the presence of infection in sika deer farmers to prevent the potential spread of the infection to both deer populations and human communities.

Initially, numerous studies showed that ST10 and ST14 were primarily found in herbivores, such as sheep, Tibetan antelope, cattle, and camels, with little evidence of human infections (17). However, a report from Senegal reported the first detection of ST10 and ST14 infections in schoolchildren (42). Additionally, Noradilah et al. found the presence of ST10 in river water in Malaysia, indicating a potential for waterborne transmission of ST10 (43). Therefore, enhanced environmental control measures around deer farms should be implemented to prevent the widespread transmission of *Blastocystis* sp. Jenny et al. were the first to report the presence of ST21, ST23, ST24, and ST26 in cervida, a finding consistent with our results (35).

In this study, five ST30-positive samples were detected. ST30 was first found and named in white-tailed deer in the United States and was later reported in Tibetan sheep, and dogs (18, 35, 44). These findings imply that ST30 have a host adaptation that enables it to spread across species. ST42 has been reported in cattle and snakes (38, 45). To our knowledge, this study is the first to report the presence of ST42 in sika deer, expanding the host range of this subtype. The detection of both ST30 and ST42 in sika deer further indicates that these subtypes specifically infect herbivores.

Although this study presents important findings, several limitations need to be addressed. First, samples were only collected during summer and autumn, which may not fully capture seasonal variations in the infection rate of *Blastocystis* sp. Future research should incorporate year-round sampling to better assess the temporal dynamics. Second, the sample size in some regions was relatively small, which may affect the representativeness of the infection rates. Larger-scale studies with more balanced sample sizes across different regions are needed to validate these findings. Moreover, this study did not account for key farm-related factors, such as farm size, stocking density, and whether the farmed sika deer were entirely captive-bred or included individuals sourced from the wild or other farms. These factors could influence infection rate dynamics, yet their absence precludes a more detailed analysis of the role of farm conditions in *Blastocystis* sp. transmission. Future studies should integrate these factors to provide a more comprehensive epidemiological assessment. Additionally, the health status of the animals was not considered, despite its potential relevance to infection susceptibility. Incorporating clinical assessments and immune profiling in future studies would offer valuable insights into host-pathogen interactions. Addressing these limitations, future studies will enhance the reliability and applicability of the findings.

## Conclusion

In summary, this study characterized that the overall infection rate of *Blastocystis* sp. was 65.02% in farmed sika deer in China. Ten subtypes was identified from 303 *Blastocystis* sp.-positive samples, including two zoonitic subtypes (ST1 and ST5). Notably, we first report the presence of ST42 in sika deer, further expanding the known diversity of *Blastocystis* sp. subtypes in wildlife. These findings suggest that sika deer may serve as a potential host for the zoonotic transmission of *Blastocystis* sp. It is recommended to conduct parasitic screenings for sika deer farmers and strengthen epidemic monitoring in the surrounding farm environment. In conclusion, this study provides important epidemiological data on the distribution of *Blastocystis* sp. in sika deer.

## Data Availability

The datasets presented in this study can be found in online repositories. The names of the repository/repositories and accession number(s) can be found at: https://www.ncbi.nlm.nih.gov/genbank/, PQ817670-PQ817696.

## Footnotes

^1^BLAST, http://www.ncbi.nlm.nih.gov/blast/

^2^ https://pubmlst.org/bigsdb?db=pubmlst_Blastocystis_seqdef
